# CircMTO1 inhibits liver fibrosis via regulation of miR‐17‐5p and Smad7

**DOI:** 10.1111/jcmm.14432

**Published:** 2019-05-31

**Authors:** Wei Wang, Ruiling Dong, Yong Guo, Jianan He, Chaopeng Shao, Pin Yi, Fujun Yu, Dayong Gu, Jianjian Zheng

**Affiliations:** ^1^ Department of Laboratory Medicine Shenzhen Second People's Hospital, The First Affiliated Hospital of Shenzhen University, Health Science Center Shenzhen China; ^2^ Shenzhen International Travel Health Care Center and Shenzhen Academy of Inspection and Quarantine Shenzhen Customs District Shenzhen China; ^3^ Institute of Organ Transplantation The First Affiliated Hospital of Wenzhou Medical University Wenzhou China; ^4^ Departments of Gastroenterology and Hepatology The First Affiliated Hospital of Wenzhou Medical University Wenzhou China; ^5^ Key Laboratory of Diagnosis and Treatment of Severe Hepato‐Pancreatic Diseases of Zhejiang Province The First Affiliated Hospital of Wenzhou Medical University Wenzhou China

**Keywords:** biomarker, chronic hepatitis B, circMTO1, liver fibrosis, miR‐17‐5p

## Abstract

Circular RNAs (circRNAs), often dysregulated in a variety of human diseases, participate in the initiation and development of cancers. Recently, circMTO1 (a circRNA derived from *MTO1* gene), identified as a tumor suppressor, has been shown to contribute to the suppression of hepatocellular carcinoma. The present study aimed to explore the clinical significance and roles of circMTO1 in liver fibrosis. Here, we found that serum circMTO1 was significantly down‐regulated in chronic hepatitis B (CHB) patients. Interestingly, serum circMTO1 was negatively correlated with fibrosis stages as well as HAI scores. Receiver operating characteristic curve analysis revealed that serum circMTO1 may serve as a diagnostic biomarker for liver fibrosis in CHB patients. Notably, overexpression of circMTO1 led to the suppression of transforming growth factor‐β1‐induced hepatic stellate cells (HSCs) activation. Bioinformatic analysis and luciferase activity assays indicated that circMTO1 was a target of mircoRNA‐17‐5p (miR‐17‐5p). Data from RNA pull‐down assay further confirmed that circMTO1 interacted with miR‐17‐5p. The inhibitory effects of circMTO1 on HSC activation were suppressed by miR‐17‐5p mimics. Further studies showed that Smad7 was a target of miR‐17‐5p. Moreover, circMTO1‐inhibited HSC activation was also blocked down by loss of Smad7. Taken together, we demonstrate that circMTO1 inhibits liver fibrosis via regulation of miR‐17‐5p and Smad7, and serum circMTO1 may be a novel promising biomarker of liver fibrosis.

## 1 INTRODUCTION

Liver fibrosis is characterized by an imbalance in the formation and degradation of extracellular matrix proteins. Notably, one of the major causes of liver fibrosis is viral hepatitis. More than 350 million persons worldwide are currently chronically Hepatitis B Virus (HBV) infected.^1^ There are an estimated 1.25 million HBV carriers in the United States as well as at least 93 million HBV carriers in China.^2^, ^3^ HBV infection remains a top health priority worldwide and a devastating cause of morbidity and mortality accounting for more than 6 00 000 deaths in 2013.^4^ Chronic hepatitis B (CHB) patients are at a relatively higher risk to develop long‐term sequelae such as cirrhosis, end‐stage liver disease, and hepatocellular carcinoma (HCC).^5^, ^6^ The accurate assessment of liver injury severity in CHB patients is helpful for the treatment. Liver biopsy is the current gold standard for the assessment of HBV‐induced liver injury severity and/or monitoring of CHB progression.^7^ However, liver biopsy is still not considered by most CHB patients due to its invasive operations and complications. Therefore, a novel promising biomarker for CHB patients is urgently needed.

Circular RNAs (circRNAs), featured by covalently closed loops, are a novel class of non‐coding RNAs without 5′‐3′ ends and ploy A tail.^8^, ^9^ Because of their featured structures, circRNAs are highly stable and hard to be degraded by exonuclease RNase R.^10^ In addition, circRNA expression has been shown in a tissue‐specific or cell type‐specific manner.^11^, ^12^ The characteristics of circRNAs make them ideal candidates as disease biomarkers.^13^ Currently, whether circRNAs in serum or plasma could serve as biomarkers for assessing liver fibrosis progression remains largely unknown.

Previously, the data from circRNA expression profile revealed down‐regulation of circRNA circMTO1 (a circRNA derived from mitochondrial tRNA translation optimization 1 [*MTO1*] gene, hsa_circ_0007874) in HCC tissues.^14^ CircMTO1 has been demonstrated to play an inhibitory role in HCC progression. Particularly, fluorescence in situ hybridization analysis indicated the location of circMTO1 in liver tissues. Therefore, circMTO1 may be involved in liver fibrosis and hepatic stellate cell (HSC) activation. In this study, the roles of circMTO1 in liver fibrosis and the clinical significance of serum circMTO1 in patients with liver fibrosis were explored.

## 2 MATERIALS AND METHODS

### 2.1 Patients

Sera were obtained from 360 therapy‐naive patients with CHB who undergone liver biopsy for staging and grading of liver fibrosis in the First Affiliated Hospital of Wenzhou Medical University between 2007.1 and 2016.3. In the same period, sera from 360 healthy controls were collected. Inclusion and exclusion criteria for CHB patients were shown in Table S1. Clinical pathological information such as age, gender, virology, alanine aminotransferase (ALT) value, Fibrosis stage and histological activity index (HAI) score was shown in Table S2. In addition, 80 serum samples of HCC were obtained from patients during operation. HCC cell lines including HepG2 and SMMC‐7721 were cultured in DMEM with 10% fetal bovine serum as routine.^15^

### 2.2 Consent statement

The informed consent was obtained from all subjects in this study and the use of serum specimens was approved by the Ethics Committee of the First Affiliated Hospital of Wenzhou Medical University. All procedures followed were in accordance with the Helsinki Declaration of 1975, revised in 1983.

### 2.3 Liver histology

Liver biopsy was performed using a 16‐gauge Menghini needle. Each liver biopsy case was advised by physicians in care, and liver specimens at least in 2.0 cm in length obtained. Samples were fixed in formalin, embedded in paraffin, and stained with hematoxylin‐eosin. Results were reviewed by experienced hepatopathologists.^16^ In addition, at least 8‐10 portal tracts in samples were required to admit patients. HAI and fibrosis stages (F0 = no fibrosis ‐ F6 = cirrhosis) were assessed using the Ishak scoring system.^17^

### 2.4 Blood sampling

After liver biopsy, the venous blood (5 mL) was obtained from each patient. Blood samples were centrifuged at 3400 g for 7 minutes at room temperature, and then the sera were collected in new Eppendorf tubes followed by further centrifugation at 12 000 g for 10 minutes at 4°C. All extracted sera were stored at −80°C pending further processing.

### 2.5 Virology

The Artus HBV QS‐RGQ Kit (Qiagen, Hilden, Germany) was performed to evaluate the level of serum HBV DNA. The Roche Modular E170 Immunoassay Analyzer (Roche, Basel, Switzerland) was used to detect the levels of HBsAg, HBeAg, and antibodies against HBsAg (anti‐HBs), HBeAg (anti‐HBe) and hepatitis B core antigen (anti‐HBc).

### 2.6 Cell culture

LX‐2 cells were cultured in DMEM supplemented with 10% fetal bovine serum and 1% penicillin‐streptomycin at 37°C and 5% CO_2_. Cells were treated with transforming growth factor‐β1 (TGF‐β1) (Sigma, St Louis, MO, USA) for 24 hours and then transduced with adenoviral vectors expressing circMTO1 (Ad‐circMTO1) (GenePharma biotechnology, Shanghai, China) or adenoviral vectors expressing a control scrambled sequence (Ad‐Ctrl) for additional 24 hours.

### 2.7 Quantitative real‐time PCR (qRT‐PCR)

TRIzol reagent (Invitrogen, Karlsruhe, Germany) and TRIzol LS Reagent (Invitrogen) were performed to extract the total RNA from LX‐2 cells and serum samples, respectively. Complementary DNA was synthesized using the ReverTra Ace qPCR RT Kit (Toyobo, Osaka, Japan). Cytoplasmic and nuclear RNA purification kits (Norgen, Thorold, Canada) were used to isolate cytoplasmic or nuclear circMTO1.^18^ Subsequently, SYBR Green real‐time PCR Master Mix (Toyobo, Osaka, Japan) was performed to measure gene expression via real‐time PCR. As described previously, the primers of circMTO1, alpha‐1 (I) collagen (Col1A1), α‐smooth muscle actin (α‐SMA) and GAPDH were designed.^14^, ^19^ The primers used for Smad7 were 5′‐CTCGGACAGCTCAATTCGGA‐3′ and 5′‐CAGTGTGGCGGACTTGATGA‐3′. TaqMan MicroRNA Assays (Applied Biosystems, Foster City, CA) were performed to detect 21 miRNAs expressions. The relative abundance of circMTO1 and mRNAs was normalized by GAPDH (Applied Biosystems, Foster City, CA) while the levels of miRNAs were normalized by U6 snRNA (Applied Biosystems, Foster City, CA). The expression level of circMTO1 in sera was calculated using ΔCt method, where ΔCt = Ct_target_–Ct_reference_, smaller ΔCt value indicates higher expression. The levels of circMTO1, mRNAs, and miRNAs in LX‐2 cells were calculated using 2^–ΔΔCt^ method.

### 2.8 Western blot analysis

Cells were lysed by radioimmunoprecipitation buffer, and then cell lysates were separated by SDS‐PAGE. After blocking, the membrane was incubated with primary antibodies (Smad7 and GAPDH, Abcam) and the secondary antibodies (Rockland, Limerick, PA, USA). Protein levels were normalized to that of total GAPDH.

### 2.9 CCK‐8 assay

LX‐2 cells at a density of 1 × 10^3^ cells per well were seeded in 96‐well plates overnight, and then treated with 2 ng/mL TGF‐β1 for 24 hours followed by the transduction of Ad‐circMTO1 for additional 24 hours. Subsequently, absorbance was measured by a microplate reader at 450 nm. The total evaluation of cell proliferation was performed by the CCK‐8 Kit (Dojindo, Kumamoto, Japan).

### 2.10 Cell cycle analysis

Cell cycle was quantified using the Cell Cycle Analysis Kit (Beyotime, China) according to the manufacturer's instruction and performed using a BD LSR flow cytometer (BD Biosciences).

### 2.11 RNA‐binding protein immunoprecipitation (RIP) assay

The EZ‐Magna RIP Kit (Millipore) was used to analyse RIP experiment. Primary HSCs lysed by RIP lysis buffer were incubated with anti‐Argonaute‐2 (Ago2) antibody. In addition, cells treated with isotype‐matched IgG were defined as the negative control group. After samples were immunoprecipitated with proteinase k, RNA was isolated and then circMTO1 level in the precipitates was quantified by qRT‐PCR.

### 2.12 Pull‐down assay

Biotin pull‐down was performed as previously described.^20^, ^21^ Briefly, cells were transfected with biotinylated miR‐17‐5p (Bio‐miR‐17‐5p), Bio‐miR‐17‐5p‐Mut, or Bio‐miR‐NC for 24 hours, and then lysed in lysis buffer. RNase‐free bovine serum albumin and yeast tRNA (Sigma) were used to remove RNA and protein complexes from the beads. Subsequently, the incubation between the lysates and streptavidin‐coated magnetic beads (Life Technologies) was performed at 4°C for 4 hours. After washing, TRIzol reagent (Life Technologies) was used to isolate the bound RNAs and the quantification of circMTO1 was determined by qRT‐PCR.

### 2.13 Luciferase reporter assay

Using lipofectamine, pmirGLO‐circMTO1 was cotransfected with the predicted miRNAs or miR‐NC into HEK293T cells as described previously.^22^ After transfection for 48 hours, the relative luciferase activity was normalized to Renilla luciferase activity.

### 2.14 Statistical analysis

Data from at least three independent experiments were expressed as the mean ± SD. Data were analysed using SPSS 13.0 (IBM, Armonk, NY). The Mann‐Whitney test or Kruskal‐Wallis test was performed to determine the significance of serum circMTO1 levels. Receiver operating characteristic (ROC) curve was generated to classify patients in different groups, as well as for the evaluation of the diagnostic potential of serum circMTO1 via calculation of the area under the ROC curve (AUC), sensitivity and specificity according to standard formulas. Correlation coefficients (r) were calculated using Spearman correlation. Differences between multiple groups in LX‐2 cells were evaluated using one‐way analysis of variance. Differences between two groups in LX‐2 cells were compared using a Student's *t*‐test. Survival curves were plotted using the Kaplan–Meier method and analysed using the log‐rank test. Data were considered significant at *P* < 0.05.

## 3 RESULTS

### 3.1 Down‐regulation of serum circMTO1 is correlated with liver fibrosis progression

**Figure 1:**
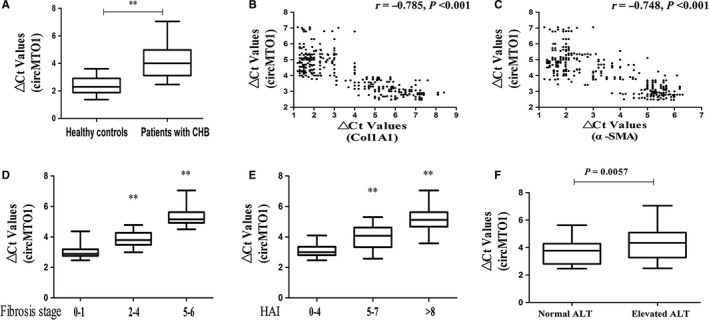
Serum circMTO1 levels were down‐regulated in chronic hepatitis B (CHB) patients and correlated with fibrosis markers. A, △Ct values of serum circMTO1 levels in CHB patients and healthy controls. B, Correlation between serum circMTO1 levels and the mRNA level of Col1A1. C, Correlation between serum circMTO1 levels and the mRNA level of α‐smooth muscle actin. D, △Ct values of serum circMTO1 levels in CHB patients with fibrosis score 0‐1, fibrosis score 2‐4 and fibrosis score 5‐6. E, △Ct values of serum circMTO1 levels in CHB patients with HAI score 0‐4, HAI score 5‐7 and HAI score >8. F, △Ct values of serum circMTO1 levels in CHB patients with different ALT values. △Ct method was used to calculate circMTO1 expression, which was normalized to GAPDH, and smaller ΔCt value indicated higher expression. Vertical lines indicate the range, and horizontal boundaries of the boxes represent the first and third quartiles. **P < 0.001 compared with the control

Due to down‐regulation of circMTO1 has been reported in HCC tumor tissues as well as HCC cell lines, whether serum circMTO1 is abnormally expressed in CHB patients was investigated. 360 CHB patients and 360 healthy controls were recruited in this study (Table S2). Between CHB patients and healthy controls, no significant difference was observed in age (*P* = 0.632) as well as sex distribution (*P* = 0.172, χ^2^ test). QRT‐PCR was performed to analyse the expression of circMTO1 in CHB patients and healthy controls. In comparison with healthy controls, serum circMTO1 expression was significantly decreased in CHB patients (Figure 1A). Next, we explored the correlation between serum circMTO1 and the markers of liver fibrosis such as Col1A1 and α‐SMA. Our data indicated that serum circMTO1 level was negatively correlated with Col1A1 mRNA level (r = −0.785, *P* < 0.001, Figure 1B). In line with it, serum circMTO1 level was additionally negatively correlated with serum α‐SMA mRNA level (r = −0.748, *P* < 0.001, Figure 1C). Taken together, down‐regulation of serum circMTO1 is negatively correlated with markers of liver fibrosis, supporting its potential utility as a biomarker for liver fibrosis. Subsequently, all CHB patients were divided into three groups according to the fibrosis scores: low‐score group (0‐1), medium‐score group (2‐4) and high‐score group (5‐6). It was found that with the increasing fibrosis scores, serum circMTO1 was significantly down‐regulated (Figure 1D). Similarly, with the increasing HAI scores, lower serum circMTO1 was observed (Figure 1E). Moreover, in comparison with patients with normal ALT, there was lower circMTO1 expression in those with elevated ALT (Figure 1F), indicating that serum circMTO1 expression was associated with inflammation and liver damage. Based on these, circMTO1 is reduced in CHB patients and negatively correlated with liver fibrosis progression.

### 3.2 The potential diagnostic value of serum circMTO1 in liver fibrosis

**Figure 2:**
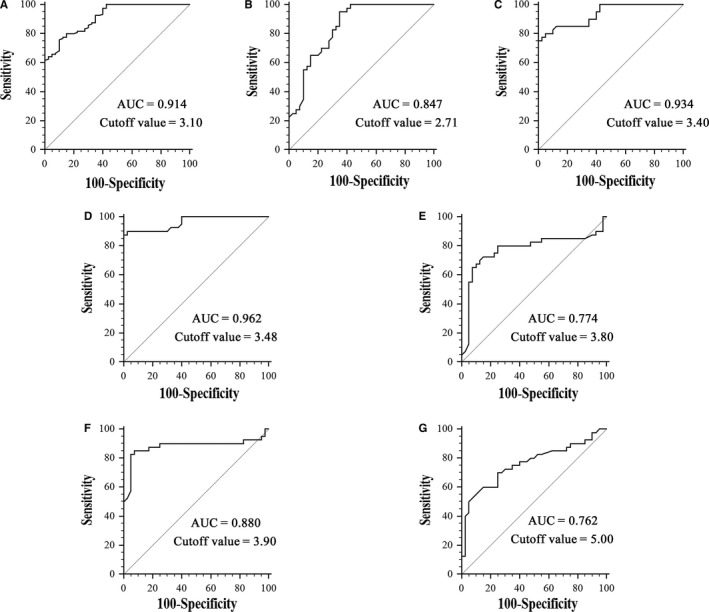
Receiver operating characteristic curve analysis of serum circMTO1 for discriminating. A, Chronic hepatitis B (CHB) patients from healthy controls. B, CHB patients with low fibrosis scores from healthy controls. C, CHB patients with medium fibrosis scores from healthy controls. D, CHB patients with high fibrosis scores from healthy controls. E, CHB patients with medium fibrosis scores from CHB patients with low fibrosis scores. F, CHB patients with high fibrosis scores from CHB patients with low fibrosis scores. G, CHB patients with high fibrosis scores from CHB patients with medium fibrosis scores

In CHB patients, the diagnostic value of serum circMTO1 for liver fibrosis was evaluated by ROC curve analysis. ROC curve analysis indicated that serum circMTO1 could effectively differentiate patients with liver fibrosis from healthy controls, with an AUC of 0.914 (95% confidence interval [CI], 0.860 to 0.953) (Figure 2A). Sensitivity was 75.8% and specificity was 90.0% at a cutoff value of 3.10. We also found that it yielded an AUC of ROC of 0.847 (95% CI 0.749 to 0.918) with 95% sensitivity and 65% specificity in discriminating CHB patients with low fibrosis scores from healthy controls (Figure 2B), an AUC of ROC of 0.934 (95% CI 0.856 to 0.977) with 80% sensitivity and 95% specificity in discriminating CHB patients with medium fibrosis scores from healthy controls (Figure 2C), and an AUC of ROC of 0.962 (95% CI 0.893 to 0.992) with 90% sensitivity and 97.5% specificity in discriminating CHB patients with high fibrosis scores from healthy controls (Figure 2D). In addition, the diagnostic value of serum circMTO1 in CHB patients with different fibrosis scores was explored. ROC analysis showed that it yielded an AUC of ROC of 0.774 (95% CI 0.667 to 0.860) with 67.5% sensitivity and 90% specificity in discriminating medium fibrosis scores from low fibrosis scores (Figure 2E), and an AUC of ROC of 0.880 (95% CI 0.788 to 0.942) with 85% sensitivity and 92.5% specificity in discriminating high fibrosis scores from low fibrosis scores (Figure 2F). Moreover, it yielded an AUC of ROC of 0.762 (95% CI 0.654 to 0.850) with 52.5% sensitivity and 92.5% specificity in discriminating high fibrosis scores from medium fibrosis scores (Figure 2G).

### 3.3 Serum circMTO1 is not associated with viral replication and liver function

**Figure 3:**
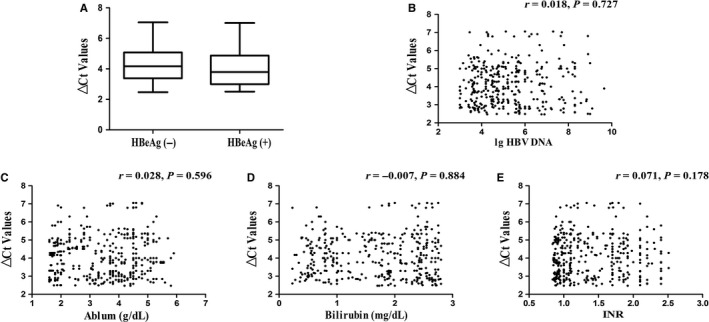
The relation between serum circMTO1 levels and hepatitis B virus (HBV) replication as well as markers of liver function. A, △Ct values of serum circMTO1 levels in chronic hepatitis B (CHB) patients with HBeAg (+) or HBeAg (―). B, Correlation between serum circMTO1 levels and HBV DNA levels in CHB patients. Correlation between serum circMTO1 levels and serum albumin concentration (C), serum bilirubin concentration (D) and INR (E)

CHB patients were additionally divided into HBeAg‐negative or HBeAg‐positive group, and then whether serum circMTO1 was associated with HBV replication was explored. However, there was no significant change in circMTO1 expression (*P* > 0.05, Figure 3A). Next, the association between serum circMTO1 and the viral load was explored. In line with the previous results, serum circMTO1 expression was not associated with HBV DNA level in CHB patients (r = 0.018, *P* = 0.727, Figure 3B). Liver function is an indicator for evaluating liver damage. Our results showed that no correlation between serum circMTO1 and the parameters of liver function including serum albumin concentration, serum bilirubin concentration and international normalized ratio (INR) was observed in CHB patients (*P* > 0.05, Figure 3C‐E). In sum, our results suggest that serum circMTO1 expression is not associated with viral replication or overall liver function.

### 3.4 Restoring of circMTO1 results in HSC inactivation

**Figure 4:**
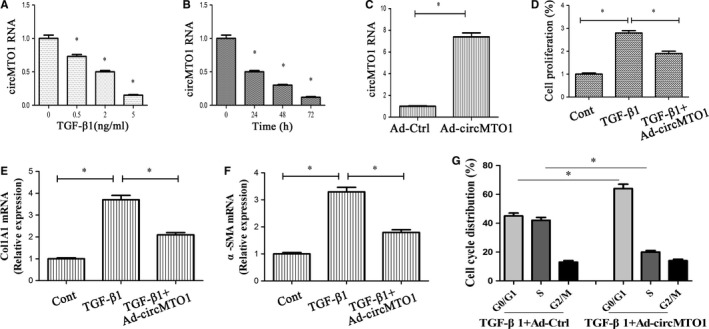
Expressions and roles of circMTO1 in transforming growth factor‐β1 (TGF‐β1)‐treated hepatic stellate cells. LX‐2 cells were treated with 2 ng/mL TGF‐β1 for 24 hours and then transduced with Ad‐circMTO1 for additional 24 hours. A, LX‐2 cells were treated with TGF‐β1 (0, 0.5, 2, and 5 ng/mL) for 24 hours. B, LX‐2 cells were treated with TGF‐β1 (2 ng/mL) for 0, 24, 48, and 72 hours. C, CircMTO1 expression. D, Cell proliferation. E, Col1A1 mRNA level. F, α‐smooth muscle actin mRNA level. G, Cell cycle. *P < 0.05 compared with the control

The activation of HSCs plays a crucial role in the initiation and development of liver fibrosis. TGF‐β1 is known as the main stimuli factor responsible for HSC activation. Next, the effects of TGF‐β1 on circMTO1 expression in LX‐2 cells were examined. TGF‐β1 treatment induced a significant reduction in circMTO1 level in a dose‐dependent manner (Figure 4A). In addition, circMTO1 expression was analysed at 0, 24, 48, and 72 hours in TGF‐β1‐treated HSCs. CircMTO1 was gradually down‐regulated during the culture period, clearly indicating reduced levels of circMTO1 in TGF‐β1‐activated HSCs (Figure 4B). Next, Ad‐circMTO1 was transduced into LX‐2 cells and induced an increase in circMTO1 expression (Figure 4C). We examined cell proliferation, collagen expression and HSC transdifferentiation in TGF‐β1‐treated cells after Ad‐circMTO1 treatment. Of note, TGF‐β1‐induced HSC activation was inhibited by circMTO1, with a reduction in cell proliferation and the expressions of Col1A1 and α‐SMA (Figure 4D‐F). Moreover, our results showed that circMTO1 overexpression inhibited cell cycle in TGF‐β1‐treated cells, associated with increased G0/G1 phase cells and reduced S phase cells (Figure 4G). These data support an inhibitory role of circMTO1 in liver fibrosis.

### 3.5 CircMTO1 is a target of miR‐17‐5p

**Figure 5:**
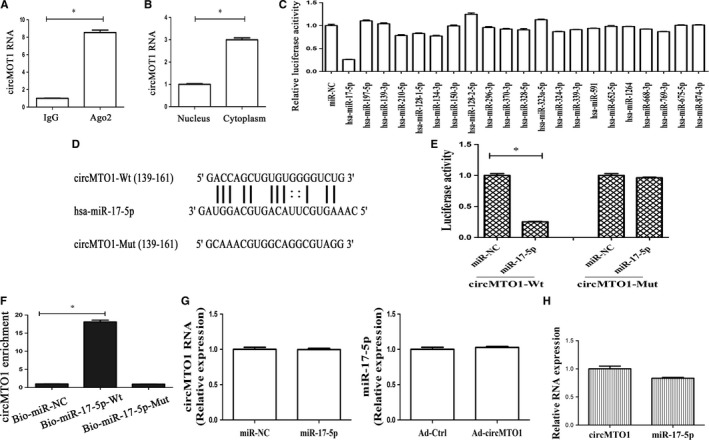
CircMTO1 acts as a binding platform for miRNAs. A, RIP experiments were performed using Ago2 antibody on extracts from LX‐2 cells. Relative level of circMTO1 was expressed as fold enrichment in Ago2 relative to IgG immunoprecipitates by qRT‐PCR. B, Expression of circMTO1 in the cytoplasm and nucleus in LX‐2 cells. C, The luciferase activity of pmirGLO‐circMTO1‐Wt in HEK‐293T cells after co‐transfection with the indicated 21 miRNAs or miR‐NC. D and E, The putative‐binding sites of miR‐17‐5p in circMTO1. Relative luciferase activities of circMTO1‐Wt or circMTO1‐Mut were analysed in HEK‐293T cells after co‐transfection with miR‐17‐5p or miR‐NC. F, Interaction between circMTO1 and miR‐17‐5p was validated by Pull‐down assay. Bio‐miR‐NC is not complementary to circMTO1. G, CircMTO1 expression in cells after miR‐17‐5p transfection and miR‐17‐5p level in circMTO1‐overexpressing cells. H, Relative expression of circMTO1 and miR‐17‐5p. *P < 0.05

Recent studies have demonstrated that circRNAs could act as miRNA sponges that regulate gene expression.^23^ To determine whether circMTO1 interacts with miRNAs, RIP experiment was performed. Results of RIP showed that circMTO1 was significantly enhanced in the Ago2 group in comparison with the control (Figure 5A). Additionally, the location of circMTO1 in HSCs was explored. CircMTO1 was found to be mainly in the cytoplasm (Figure 5B). MiRanda miRNA target prediction tool was used to find 21 potential miRNAs that may bind to circMTO1 (Figure 5C). Then, luciferase activity assays were performed to confirm it. Among these miRNA candidates, miR‐17‐5p was shown to down‐regulate the luciferase reporter activity by at least 70% (Figure 5C). Meanwhile, the activity of luciferase reporter with mutated sites was not affected by miR‐17‐5p (Figure 5D and 5E). As shown by RNA pull‐down assay, enhanced circMTO1 expression was found in bio‐miR‐17‐5p group (Figure 5F). CircMTO1 did not show significant changes after overexpression of miR‐17‐5p. Similarly, miR‐17‐5p showed no significant changes after overexpressing circMTO1 (Figure 5G). In LX‐2 cells, circMTO1 expression was higher than miR‐17‐5p level (Figure 5H). Combined with these, we demonstrate that circMTO1 could serve as a sponge for miR‐17‐5p.

### 3.6 CircMTO1 suppresses HSC activation via miR‐17‐5p and Smad7

**Figure 6:**
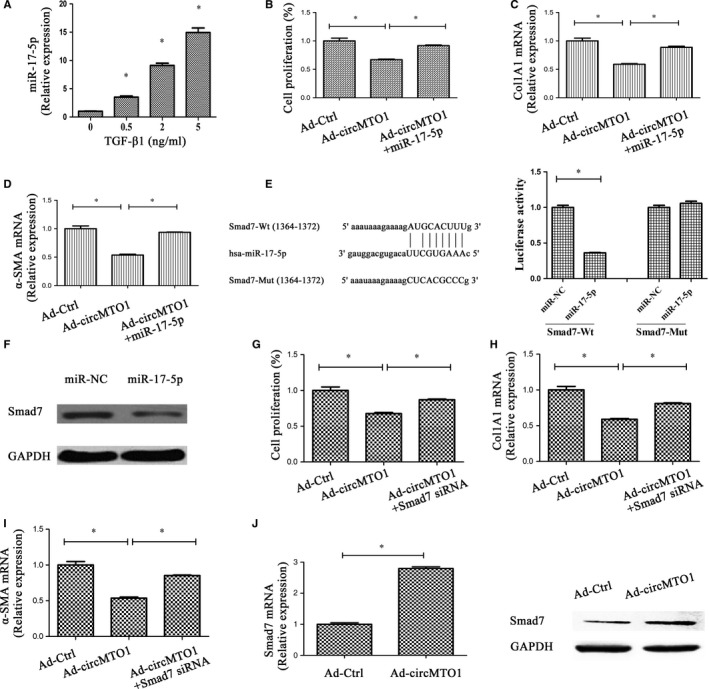
CircMTO1 inhibits activation of hepatic stellate cells via miR‐17‐5p and Smad7. Cells were transduced with Ad‐circMTO1 for 24 hours and then transfected with miR‐17‐5p mimics or Smad7 siRNA for additional 24 hours. A, Expression of circMTO1 in transforming growth factor‐β1‐treated cells. Cell proliferation (B), and expressions of Col1A1 (C) and α‐smooth muscle actin (D) were suppressed by Ad‐circMTO1 treatment, which was blocked down by miR‐17‐5p. E, The putative‐binding sites of miR‐17‐5p in Smad7. Relative luciferase activities of Smad7‐Wt or Smad7‐Mut were analysed in HEK‐293T cells after co‐transfection with miR‐17‐5p or miR‐NC. F, Smad7 protein was inhibited by miR‐17‐5p. Ad‐circMTO1‐inhibited cell proliferation (G), Col1A1 (H) and α‐SMA (I) were restored by Smad7 siRNA. J, Smad7 was increased by circMTO1 overexpression. *P < 0.05

To determine whether miR‐17‐5p plays a crucial role in the inhibitory effects of circMTO1 on HSC activation, we firstly examined miR‐17‐5p expression in cells after TGF‐β1 treatment. MiR‐17‐5p was shown to be increased by TGF‐β1 in a dose‐dependent manner (Figure 6A). Next, circMTO1‐overexpressing cells were transfected with miR‐17‐5p mimics. Interestingly, circMTO1‐suppressed HSC activation including proliferation, Col1A1 and α‐SMA was almost restored by miR‐17‐5p (Figure 6B‐D). Previously, it has been reported that miR‐17‐5p contributes to liver fibrosis via its target Smad7 in rats.^24^ Accordingly, Smad7 was predicted and confirmed as a target of hsa‐miR‐17‐5p (Figure 6E). As shown in Figure 6F, miR‐17‐5p led to a reduction in Smad7 protein. To determine whether Smad7 was involved in the effects of circMTO1 on HSC inactivation, cell proliferation, Col1A1 and α‐SMA were examined in circMTO1‐overexpressing cells after Smad7 siRNA transfection. Our results showed that Ad‐circMTO1‐inhibited HSC activation was restored by Smad7 siRNA (Figure 6G‐I), which was similar with the results of circMTO1‐overexpressing cells after miR‐17‐5p treatment. Notably, Smad7 mRNA level was increased by circMTO1 overexpression (Figure 6J). In line with the mRNA result, immunoblot assays confirmed that Smad7 protein was enhanced by circMTO1 overexpression. All the findings suggest that circMTO1 inhibits HSC activation via miR‐17‐5p and Smad7.

### 3.7 CircMTO1/miR‐17‐5p/Smad7 axis is existed in HCC and low serum circMTO1 is correlated with poor prognosis

We additionally examined the expression of serum circMTO1 in HCC patients. Obviously, serum circMTO1 in patients with HCC was lower than that in healthy controls and patients with CHB (Figure S1). Next, the association between circMTO1 expression and HCC prognosis was analysed using Kaplan‐Meier analysis and log‐rank test. It was found that HCC patients with high expression of circMTO1 had a higher survival rate than those with low circMTO1 (median overall survival, 38.5 months vs 25.2 months, *P* < 0.0001) (Figure S2). Interestingly, circMTO1/miR‐17‐5p/Smad7 signalling could be found in HCC. As shown in Figure S3, reduced HCC cell proliferation caused by circMTO1 was restored by miR‐17‐5p or Smad7 siRNA. These data suggest that circMTO1/miR‐17‐5p/Smad7 axis is also existed in HCC and serum circMTO1 may be a potential biomarker in HCC.

## 4 DISCUSSION

Dysregulation of circRNAs has been found in various human diseases including cancers.^25^ Increasing evidence has reported the involvement of circRNAs in vital biological processes such as proliferation, metastasis and therapy resistance.^26^ Current studies are mainly focused on biology functions of circRNAs, whereas little is known about their clinical diagnostic value in human diseases. In fact, the detection of circRNAs in plasma has been shown to be abundant and stable.^27^ Particularly, blood‐based biomarkers are non‐invasive and more easily accepted by patients. Increasing studies support serum non‐coding RNA utility as a potential biomarker for the diagnosis of diseases.^28^, ^29^ Due to highly conserved sequences and a high degree of stability, circRNAs may be superior to miRNAs and long non‐coding RNAs in the diagnosis of human diseases. In this study, serum circMTO1 was detected in CHB patients and its clinical significance was evaluated. Consistent with the findings in HCC,^14^ down‐regulation of serum circMTO1 level was observed in liver fibrosis. A negative correlation between serum circMTO1 and liver fibrosis stages as well as liver injury degree was observed. ROC analysis revealed a significant diagnostic value of liver circMTO1 for liver fibrosis in CHB patients. Additionally, serum circMTO1 could be discriminated in CHB patients with different fibrosis stages. Overall, serum circMTO1 may be a novel promising biomarker of liver fibrosis. Whether serum circMTO1 could be a potential biomarker for HCC patients was also examined. Our results showed that lower serum circMTO1 was found in HCC patients when compared with healthy controls and CHB patients. Further studies demonstrated that low serum circMTO1 was correlated with poor prognosis of HCC patients. Therefore, serum circMTO1 may be a potential biomarker for HCC patients and further studies are needed to prove it.

The expression and role of circMTO1 in TGF‐β1‐induced HSC activation were determined in this study. It was found that circMTO1 was down‐regulated in a dose‐dependent and time‐dependent manner in TGF‐β1‐treated HSCs. Of note, TGF‐β1‐casued HSC activation, characterized by changes in cell proliferation, collagen expression and HSC transdifferentiation, was inhibited by circMTO1. A growing body of evidence suggests that circRNAs may participate in disease progression via serving as miRNA sponges to protect the target genes from miRNA‐induced mRNA cleavage.^23^ In this study, we found that circMTO1 could act as a binding platform for miRNAs. Using bioinformatic analysis and luciferase activity assays, circMTO1 was found to interact with miR‐17‐5p. This interaction was directly confirmed by the RNA pull‐down assay. Moreover, circMTO1 and miR‐17‐5p could not be digested by each other. Taken together, our results suggest that circMTO1 could serve as a sponge for miR‐17‐5p.

MiR‐17‐5p serves as an oncogenic miRNA to contribute to cancer development.^30^, ^31^ Numerous studies have already shown that miR‐17‐5p could modulate cell cycle to promote cell proliferation.^32^ Up‐regulation of serum miR‐17‐5p indicates a poor prognosis in various human cancers.^33^, ^34^ Consistently, miR‐17‐5p plays a pro‐fibrotic role in liver fibrosis.^35^ Previously, our group demonstrated that up‐regulation of miR‐17‐5p led to HSC activation through Smad7.^24^ Smad7 is known as a negative regulator in TGF‐β/Smads pathway. Herein, we demonstrated the involvement of miR‐17‐5p and Smad7 in the effects of circMTO1 on HSC activation. CircMTO1‐suppressed HSC activation was restored by miR‐17‐5p mimics or Smad7 knockdown. The similar results could also be found in HCC, indicating the existence of circMTO1/miR‐17‐5p/Smad7 signalling axis in liver diseases. Taken together, circMTO1 suppresses liver fibrosis progression through regulation of miR‐17‐5p and Smad7.

In conclusion, circMTO1 suppresses the progression of liver fibrosis via regulation of miR‐17‐5p and Smad7. Moreover, serum circMTO1 is a novel promising biomarker of liver fibrosis.

## CONFLICT OF INTEREST

The authors confirm that there are no conflicts of interest.

## AUTHORS’ CONTRIBUTIONS

Wei Wang, Ruiling Dong, Yong Guo and Jianan He carried out most of the experiments; Chaopeng Shao and Pin Yi analysed the data; Fujun Yu, Dayong Gu and Jianjian Zheng provided the statistical support and wrote the manuscript. All authors have read the manuscript and approved the final version.

## Data Availability

All data generated or analysed during this study are included in this article.
