# Supplementary material for: CircMTO1 inhibits liver fibrosis via regulation of miR‐17‐5p and Smad7
Source: J Cell Mol Med. 2019 May 31;23(8):5486–96. doi: 10.1111/jcmm.14432 (PMC6653252; doi:10.1111/jcmm.14432)
Supplement: Supplementary file 1 [file JCMM-23-5486-s001.doc]

**Table S1 Inclusion and exclusion criteria for CHB patients**

| Criteria | Inclusion | exclusion |
| --- | --- | --- |
| 1. detectable serum HBs antigen and serum HBV DNA for more than six months | √ |  |
| 1. patients aged less than 16 years |  | √ |
| 1. co-infection with human immunodeficiency virus |  | √ |
| 1. coexistence of liver injury caused by other etiologies, including hepatitis C virus (HCV) infection, drug intake, alcohol consumption and auto-immune hepatitis |  | √ |
| 1. severe systematic diseases |  | √ |
| 1. pregnancy and lactation |  | √ |
