# Supplementary material for: CircMTO1 inhibits liver fibrosis via regulation of miR‐17‐5p and Smad7
Source: J Cell Mol Med. 2019 May 31;23(8):5486–96. doi: 10.1111/jcmm.14432 (PMC6653252; doi:10.1111/jcmm.14432)
Supplement: Supplementary file 2 [file JCMM-23-5486-s002.doc]

**Table S2 Patient Characteristics**

| Parameter | CHB patients | healthy subjects |
| --- | --- | --- |
| *Epidemiology* |  |  |
| Age, years, median (range) | 46.6 (31.4-57.3) | 48.0 (29.9-58.8) |
| Gender, m/f (%) | 212/148 (58.9/41.1) | 188/172 (52.2/47.8) |
| *Virology* |  |  |
| HBe antigen negative, n (%) | 185 (51.4%) |  |
| HBe antigen positive, n (%) | 175 (48.6%) |  |
| *ALT* |  |  |
| Normal ALT | 125 (34.7%) |  |
| Elevated ALT* | 235 (65.3%) |  |
| *Fibrosis stage (Ishak)* |  |  |
| F0, n (%) | 33 (9.2%) |  |
| F1, n (%) | 30 (8.3%) |  |
| F2, n (%) | 68 (18.9%) |  |
| F3, n (%) | 72 (20.0%) |  |
| F4, n (%) | 64 (17.8%) |  |
| F5, n (%) | 62 (17.2%) |  |
| F6, n (%) | 31 (8.6%) |  |
| *HAI* |  |  |
| 2, n (%) | 24 (6.7%) |  |
| 3, n (%) | 48 (13.3% ) |  |
| 4, n (%) | 43 (11.9%) |  |
| 5, n (%) | 53 (14.7%) |  |
| 6, n (%) | 49 (13.6% ) |  |
| 7, n (%) | 40 (11.1% ) |  |
| 8, n (%) | 31 (8.6%) |  |
| 9, n (%) | 44 (12.2% ) |  |
| ≥11, n (%) | 28 (7.8%) |  |

*>40 U/L.
