# Supplementary material for: CircMTO1 inhibits liver fibrosis via regulation of miR‐17‐5p and Smad7
Source: J Cell Mol Med. 2019 May 31;23(8):5486–96. doi: 10.1111/jcmm.14432 (PMC6653252; doi:10.1111/jcmm.14432)
Supplement: Supplementary file 3 [file JCMM-23-5486-s003.docx]

**Supporting Information**

**Fig.S1** Serum circMTO1 levels were down-regulated in HCC patients (n=80) when compared with CHB patients (n=360) and healthy controls (n=360). △Ct method was used to calculate circMTO1 expression, which was normalized to GAPDH, and smaller ΔCt value indicated higher expression. Vertical lines indicate the range, and horizontal boundaries of the boxes represent the first and third quartiles. ^**^*P*<0.001 compared with the control.


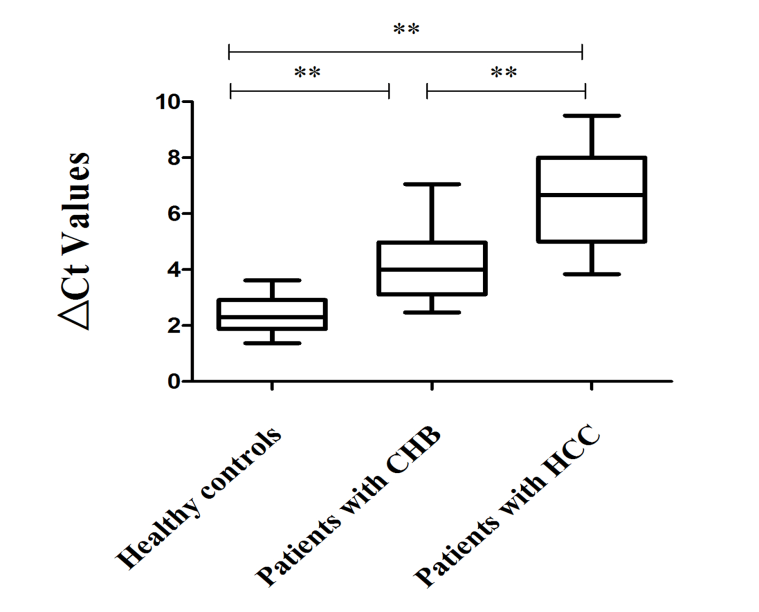


**Fig.S2** Kaplan-Meier survival for HCC patients with low or high expression of serum circMTO1.

**
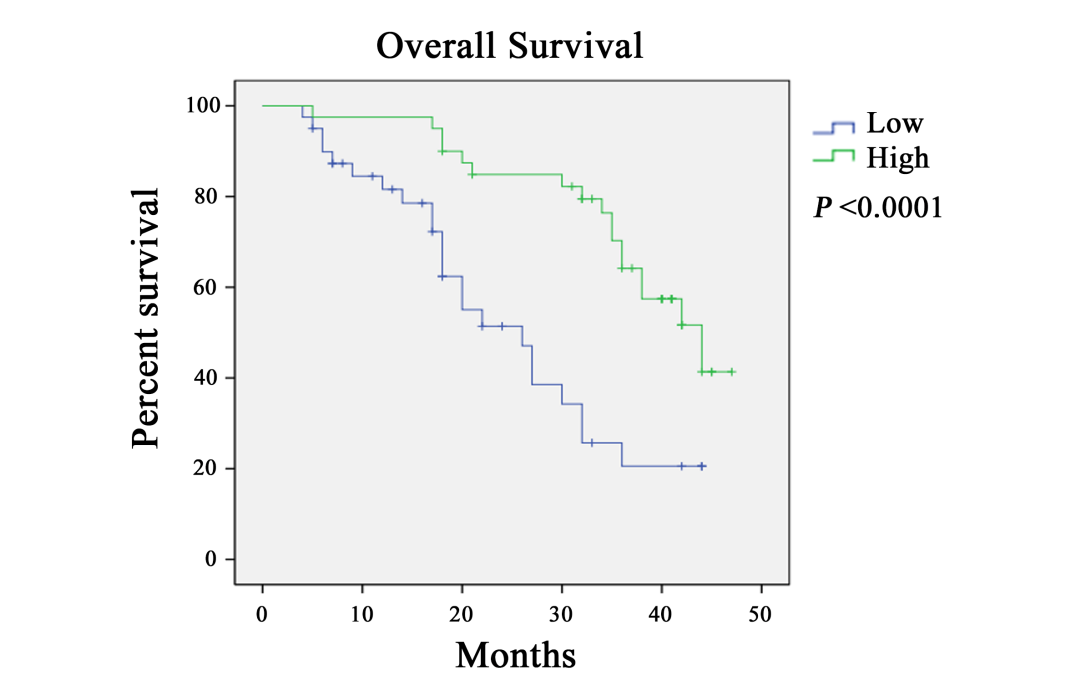
**

**Fig.S3** CircMTO1-miR-17-5p-Smad7 signaling axis in HCC. In HepG2 and SMMC-7721 cells, circMTO1 over-expression-reduced cell proliferation was inhibited by miR-17-5p or Smad7 siRNA. Cells were transduced with Ad-circMTO1 for 24 h and then treated with Smad7 siRNA or miR-17-5p mimics for additional 24 h. **P*<0.05 compared with the Ad-Ctrl group and ^#^*P*<0.05 compared with the Ad-circMTO1 group. Each value is the mean ± SD of three experiments.
